# Association of serum Spp1 levels with disease progression in ALS and SBMA

**DOI:** 10.1002/acn3.52087

**Published:** 2024-05-22

**Authors:** Woohee Ju, Jae‐Jun Ban, Hye‐ryeong Im, Sun Hi Ko, Jaewoo Seo, Young Gi Min, Yoon‐Ho Hong, Seok‐Jin Choi, Jung‐Joon Sung

**Affiliations:** ^1^ Department of Neurology Seoul National University Hospital Seoul Republic of Korea; ^2^ Department of Translational Medicine Seoul National University College of Medicine Seoul Republic of Korea; ^3^ Biomedical Research Institute Seoul National University Hospital Seoul Republic of Korea; ^4^ Department of Neurology Seoul Metropolitan Government Seoul National University Boramae Medical Center Seoul Republic of Korea; ^5^ Center for Hospital Medicine Seoul National University Hospital Seoul Republic of Korea; ^6^ Neuroscience Research Institute Seoul National University College of Medicine Seoul Republic of Korea

## Abstract

**Objective:**

In comparison with amyotrophic lateral sclerosis (ALS), the contribution of neuroinflammation in spinobulbar muscular atrophy (SBMA) has been less explored. We investigated the role of neuroinflammation in the pathogenesis of ALS and SBMA by analyzing systemic inflammatory markers and osteopontin (Spp1).

**Methods:**

This study involved 105 ALS, 77 SBMA, and 55 healthy controls. We measured their systemic inflammatory markers, serum Spp1, and cytokine levels (interferon‐γ, interleukin [IL]‐1β, IL‐6, IL‐8, IL‐10, tumor necrosis factor‐α, and IL‐17A), investigated correlations between Spp1 levels and clinical features, and evaluated ALS survival rates according to Spp1 levels.

**Results:**

In the ALS group, systemic inflammatory markers were significantly higher than in the control and SBMA groups. Spp1 levels were observed to be higher in ALS patients, but the difference was not statistically significant among the study groups. Cytokine profiles were comparable. In ALS, higher Spp1 levels were correlated with lower ALS Functional Rating Scale‐Revised (ALSFRS‐R) scores (*r* = −0.25, *p* = 0.02) and faster disease progression rate (*r* = 0.37, *p* < 0.001). After adjusting for other prognostic indicators, high Spp1 levels were independently associated with shorter survival in ALS patients (hazard ratio 13.65, 95% confidence interval 2.57–72.53, *p* < 0.01).

**Interpretation:**

Neuroinflammation does not appear to be a primary contributor to the pathogenesis of SBMA. Serum Spp1 levels may serve as a reliable biomarker for disease progression and prognosis in ALS. These findings expand our understanding of these two distinct motor neuron disorders and offer a potential biomarker for future studies.

## Introduction

Amyotrophic lateral sclerosis (ALS) and spinobulbar muscular atrophy (SBMA) are distinct neurodegenerative disorders that primarily affect motor neurons in the spinal cord and, in the case of ALS, the brain. SBMA is an X‐linked, monogenic disorder caused by an abnormally expanded CAG trinucleotide repeat in the first exon of the androgen receptor (*AR*) gene,^1^ whereas a single‐gene variant does not solely explain the pathophysiology for most cases of sporadic ALS. Instead, aging‐related dysfunction and environmental factors at least partially influence the disease pathogenesis.^2^

Motor neuron degeneration in ALS occurs both cell‐autonomously within motor neurons and non‐cell‐autonomously through the involvement of nonneuronal cells such as astrocytes and microglia in the CNS and peripheral immune cells.^3^ It is presumed that the molecular pathophysiology of ALS, which involves altered protein homeostasis, defects in RNA processing,^4^ and mitochondrial dysfunction,^5^ ultimately affects the immune system. Dysregulated immune responses are increasingly accepted as a significant contributor to disease progression.^6^ Nevertheless, although nonneuronal manifestations such as myopathy,^7^ liver pathology,^8^ and increased insulin resistance^9^ are widely recognized in SBMA, unlike ALS, the role of neuroinflammatory responses in the pathogenesis of SBMA is poorly understood.

Osteopontin (Spp1), which has been identified as a biomarker of neuroinflammation, plays an important role in immune cell recruitment and neuroglial crosstalk in the CNS.^10^ Increased levels of Spp1 in plasma and cerebrospinal fluid (CSF) have been observed in patients with chronic inflammatory and neurodegenerative disorders, such as multiple sclerosis^11^ and Alzheimer disease.^12^, ^13^ In ALS, recent studies have shown that Spp1 expression is upregulated in the spinal cord during the presymptomatic stages of ALS,^14^ and patients with high serum Spp1 levels exhibit a more severe disease state.^15^ However, the associations of serum Spp1 with detailed clinical features of ALS, as well as its prognostic value have not been extensively studied.

In this study, we aimed to investigate the systemic inflammatory profile and serum Spp1 levels in patients with ALS and SBMA, and to examine the associations of Spp1 levels with clinical variables and other systemic inflammatory markers in motor neuron diseases (MNDs).

## Methods

### Study participants

A flowchart depicting the patient selection process is displayed in Figure S1. Patients with ALS and SBMA were retrospectively identified from the MND registry of Seoul National University Hospital. Data on ALS patients were collected from February 2017 to August 2023, while data on those with SBMA were collected from January 2002 to December 2023. Patients were excluded for the following reasons: insufficient clinical and laboratory data (ALS: *n* = 279; SBMA: *n* = 75), taking oral steroids or immunosuppressants (ALS: *n* = 153; SBMA: *n* = 13), history of cancer (ALS: *n* = 16; SBMA: *n* = 6), concurrent infection (ALS: *n* = 35; SBMA: *n* = 5), and rheumatoid or autoimmune disease (ALS: *n* = *n* = 8; SBMA: *n* = 1). After applying these exclusion criteria, a total of 105 patients with ALS and 77 with SBMA were included in the final analysis. All patients with ALS met the criteria for definite, probable, or possible ALS as per the revised El Escorial criteria. All patients with SBMA harbored a pathogenic expansion of the CAG trinucleotide repeat (>38 repeats) in the *AR* gene. Additionally, data on 55 male controls were collected from the neuromuscular clinic of the Department of Neurology or the outpatient clinic of the Department of Family Medicine in chronological order.

### Standard protocol approvals, registration, and patient consents

This study was conducted in accordance with the principles of the Declaration of Helsinki and was approved by the Institutional Review Board (IRB) of Seoul National University Hospital (IRB No. 2110‐078‐1262). Due to the retrospective nature of the study, the requirement for informed consent was waived by the IRB.

### Clinical data and laboratory findings

Clinical characteristics, such as sex, age of onset (defined as the first patient‐reported symptom), age at diagnosis, onset region, ALS Functional Rating Scale‐Revised (ALSFRS‐R) score, height, weight, and comorbidities were examined. The progression rate was determined by subtracting the ALSFRS‐R score at the time of assessment from 48 and then dividing the result by the disease duration in months. The date of death was obtained from a database of the Ministry of the Interior and Safety, with the reference date of December 15, 2023. The upper and lower motor neuron signs in each anatomical segment were assessed by a board‐certified neurologist using a combination of neurological examination and electromyography findings. The results of the following blood tests were recorded: white blood cell (WBC) count (neutrophil and lymphocyte count in 10^3^/μL), creatine kinase (CK, U/L), creatinine (mg/dL), erythrocyte sedimentation rate (ESR, mm/h), and C‐reactive protein (CRP, mg/dL). The neutrophil‐to‐lymphocyte ratio (NLR) was calculated by dividing the absolute number of neutrophils by the absolute number of lymphocytes. In ALS patients, the results of pulmonary function tests, including forced vital capacity (FVC, %), forced expiratory volume in 1 second (FEV1, %), sniff nasal inspiratory pressure (SNIP, cmH_2_O), maximum inspiratory pressure (MIP, cmH_2_O), maximum expiratory pressure (MEP, cmH_2_O), and peak expiratory flow (PEF, cmH_2_O), were collected.

### Serum osteopontin (Spp1) and cytokines

In each group, we utilized available samples from patients with ALS (*n* = 88) and SBMA (*n* = 28), as well as healthy controls (*n* = 31), whose samples were stored in serum separator tubes. The blood was allowed to clot for at least 30 min, and then it was centrifuged at 1500 rpm for 10 min at 4°C. The supernatant was frozen in aliquots at −80°C until use. If available, follow‐up samples were taken at least 6 months apart (23 samples from 11 ALS patients). The serum Spp1 concentration was measured using the commercially available ELISA kit (Abcam, ab269374) according to the manufacturer's instructions. Briefly, serum was diluted at 1:30 with diluent buffer and loaded onto the plate. The antibody cocktail was added to each well and incubated at room temperature for 1 h. After washing three times with wash buffer, the TMB solution was reacted for 10 min, and stop solution was added. Absorbance at 450 nm was measured using a microplate reader (Molecular Devices). Serum cytokine levels in 14 patients with ALS, 15 with SBMA, and 10 healthy controls selected by age group were measured using the Meso Scale Discovery V‐PLEX multiplex assay kit, which included interferon (IFN)‐γ, interleukin (IL)‐1β, IL‐6, IL‐8, IL‐10, tumor necrosis factor (TNF)‐α, and IL‐17A. All samples were analyzed in duplicate by laboratory technicians who were blinded to the clinical data.

### Statistical analysis

The normality of the distribution was assessed using the Shapiro–Wilk test. Normally distributed variables were expressed as mean ± standard deviation, and non‐normally distributed variables were expressed as median (interquartile range [IQR]). Categorical variables were expressed as number (percentage). Differences between two groups were analyzed using the Welch's *t*‐test or Wilcoxon rank‐sum test for continuous variables, and the chi‐squared test for categorical variables. To compare among three groups while adjusting for covariates, analysis of covariance (ANCOVA) and multivariable linear regression analysis were conducted. The correlations between variables were assessed using the Spearman correlation test. All biomarkers were transformed using the logarithmic method for analysis. Age was adjusted in all comparative analyses.

In ALS group, the functional endpoint was defined as either death from any cause or the introduction of tracheostomy‐invasive ventilation. Kaplan–Meier curves based on serum Spp1 levels were generated and compared using the log‐rank test. The appropriate cutoff point for Spp1 was determined based on the value with the largest standardized log‐rank statistic and was calculated using the “maxstat” package in R program. Univariable and multivariable Cox proportional–hazard regression analyses were performed to assess the prognostic value of serum Spp1 concentrations. Based on previous research,^16^, ^17^ demographic and clinical characteristics such as age, sex, and disease onset, BMI, diagnostic delay, disease progression rate, ALSFRS‐R, and FVC were included as covariates. A two‐tailed *p‐*value of less than 0.05 was considered statistically significant. All statistical analyses were conducted using R version 4.3.2.

## Results

### Clinical characteristics

In total, 237 participants were included in the study, comprising 105 patients with ALS, 77 with SBMA, and 55 healthy controls (Table 1). The sex ratio differed significantly, with female patients only included in the ALS group (*n* = 35). The mean age of onset was significantly older in patients with ALS than in those with SBMA (57.5 ± 10.4 vs. 48.8 ± 9.8 years, *p* < 0.001). The median ALSFRS‐R score at diagnosis was significantly lower in ALS patients than in SBMA patients (40 [36–43] vs. 42 [39–45], *p* < 0.05). Additionally, the median disease progression rate (DPR) was significantly faster in ALS patients than in SBMA patients (0.73 [0.35–1.15] vs. 0.14 [0.08–0.20], *p* < 0.001). In SBMA patients, the median CAG repeat number was 48 [46–50]. In ALS patients, the median FVC (% predicted) was 74 [61–89].

**Table 1:** Clinical characteristics of the study participants.

	ALS (*n* = 105)	SBMA (*n* = 77)	Control (*n* = 55)	*p*‐Value			
				Overall	ALS vs. control	SBMA vs. control	ALS vs. SBMA
Sex (M/F)^a^	70/35	77/0	55/0	***	***	1.00	***
Age of onset (years)^b^	57.5 ± 10.4	48.8 ± 9.8	NA				***
Age at diagnosis (years)^b^	58.9 ± 10.3	53.5 ± 10.1	NA				***
Age at sampling (years)^b^	59.1 ± 10.7	54.1 ± 9.9	57.9 ± 10.6	**	0.78	0.09	**
BMI (kg/m^2^)	22.4 (20.8–24.5)	23.2 (21.5–24.9)	23.9 (22.7–26.0)	0.26			
Onset region^a^							
Bulbar (%)	25 (23.8)	NA	NA				
Cervical (%)	48 (45.7)	NA	NA				
Lumbosacral (%)	32 (30.5)	NA	NA				
Disease duration (months)^c^	12 (8–18)	47 (17–72)	NA				***
ALSFRS‐R at diagnosis^c^ ^,^ ^d^	40 (36–43)	42 (39–45)	NA				*
DPR^c^ ^,^ ^d^	0.73 (0.35–1.15)	0.14 (0.08–0.20)	NA				***
CAG repeat number^c^	NA	48 (46–50)	NA				
Pulmonary function test							
FVC (%)	74 (61–89)	NA	NA				
FEV_1_ (%)	80 (63–90)	NA	NA				
PEF (cmH_2_O)	230.0 (193.3–423.3)	NA	NA				
MIP (cmH_2_O)	41.8 (23.3–62.9)	NA	NA				
MEP (cmH_2_O)	47.5 (28.3–66.4)	NA	NA				
SNIP (cmH_2_O)	38.0 (23.5–55.5)	NA	NA				

Statistical analysis methods: Fisher's exact test, ANOVA, Welch's *t*‐test, or Wilcoxon rank‐sum test.

ALS, amyotrophic lateral sclerosis; ALSFRS‐R, ALS Functional Rating Scale‐Revised; BMI, body mass index; DPR, disease progression rate; F, female; FEV, forced expiratory volume; FVC, forced vital capacity; M, male; MEP, maximal expiratory pressure; MIP, maximal inspiratory pressure; NA, not applicable; PEF, peak expiratory flow; SBMA, spinobulbar muscular atrophy, SNIP, sniff nasal inspiratory pressure.

^a^ Data are expressed as the number of patients.

^b^ Data are expressed as mean ± SD.

^c^ Data are expressed as median (interquartile range).

^d^ Data were not recorded for 2 ALS and 39 SBMA patients. DPR = (48 − ALSFRS‐R at diagnosis)/time from onset to diagnosis (month).

* *p* < 0.05;

** *p* < 0.01;

*** *p* < 0.001.

### Laboratory findings and serum Spp1 concentrations

Biochemical profiles were compared among 105 patients with ALS, 77 with SBMA, and 34 healthy controls (Table 2). The age at sampling of ALS patients was significantly older than that of SBMA patients. Controlling for age at sampling as a confounding variable, inflammatory markers such as the WBC count and ESR were significantly higher in ALS than in healthy controls. Furthermore, NLR and CRP were also significantly elevated in ALS patients compared to those with SBMA. However, in patients with SBMA, the slightly elevated WBC count was the only parameter with a statistically significant difference compared to healthy controls. In the multivariable linear regression analysis, CRP and ESR remained significantly high compared to controls (Table S1). The CK levels were significantly elevated in both ALS and SBMA patients, with a more pronounced increment observed in SBMA. On the contrary, creatinine levels were significantly reduced in both ALS and SBMA patients, with a more pronounced decrement observed in the SBMA group.

**Table 2:** Comparisons of laboratory biomarkers among study groups.

	ALS	SBMA	Control	*p*‐Value for ANCOVA			
				Overall	*Post hoc* analysis		
					ALS vs. control	SBMA vs. control	ALS vs. SBMA
Age at sampling (years)^a^	59.1 ± 10.7 (*n* = 105)	54.1 ± 9.9 (*n* = 77)	57.9 ± 10.6 (*n* = 34)	**	0.78	0.09	**
WBC (10^3^/μL)^b^	6.86 (5.80–8.49)	6.29 (5.52–8.00)	5.34 (4.87–6.37)	***	***	*	0.23
NLR^b^	2.13 (1.61–2.90)	1.46 (1.18–1.98)	1.69 (1.28–2.23)	***	0.09	0.49	***
CRP (mg/dL)^b^	0.09 (0.04–0.20)	0.03 (0.01–0.11)	0.06 (0.02–0.12)	***	0.06	0.64	***
ESR (mm/h)^b^	16 (8–24)	9 (4–14)	8 (5–16)	***	**	0.99	**
CK (U/L)^b^	200 (123–344)	816 (488–1497)	183 (104–207)	***	0.66	***	***
Cr (mg/dL)^b^	0.71 (0.60–0.80)	0.60 (0.55–0.77)	0.90 (1.81–1.00)	***	***	***	*
Age at sampling (years)^a^	58.9 ± 10.3 (*n* = 88)	54.9 ± 10.8 (*n* = 28)	58.3 ± 12.3 (*n* = 31)	0.23			
Spp1 (ng/mL)^b^	23.08 (15.85–30.65)	20.90 (16.18–29.53)	20.46 (13.80–25.90)	0.22			

Statistical analysis methods: ANOVA for age at sampling and ANCOVA for biomarkers (covariate: age at sampling) were used. Tukey method was used for comparing a family of three estimates. All biomarkers have been transformed using the logarithmic method for analysis.

ALS, amyotrophic lateral sclerosis; CK, creatine kinase; Cr, creatinine; CRP, C‐reactive protein; ESR, erythrocyte sedimentation rate; NLR, neutrophil‐to‐lymphocyte ratio; SBMA, spinobulbar muscular atrophy; Spp1, secreted phosphoprotein 1; WBC, white blood cell.

^a^ Data are expressed as mean ± SD.

^b^ Data are expressed as median (interquartile range).

* *p* < 0.05;

** *p* < 0.01;

*** *p* < 0.001.

Serum Spp1 levels were measured in 88 patients with ALS, 28 with SBMA, and 31 controls. Patients' age did not significantly differ across these groups (Table 2, *p* = 0.23). While Spp1 concentrations seemed to be slightly higher in patients with ALS (23.08 [15.85–30.65] ng/mL) than in SBMA patients (20.90 [16.18–29.53] ng/mL) and healthy controls (20.46 [13.80–25.90] ng/mL), the differences were not significant (*p* = 0.22; Table 2). However, in a sensitivity analysis of male patients, Spp1 was significantly higher in patients with ALS than in healthy controls, even after adjusting for age at sampling (*p* = 0.04 and *p* = 0.01, respectively; Tables S2 and S3). The serum levels of cytokines (pro‐inflammatory: TNF‐α, IFN‐γ, IL‐1β, IL‐6, and IL‐17A; anti‐inflammatory: IL‐10) were comparable among the groups (Table S4). There were no significant correlations observed between Spp1 concentrations and other inflammatory markers (WBC, NLR, and CRP) or cytokine levels (data not shown).

### Associations between serum Spp1 and clinical features

#### Disease Severity

**Figure 1:**
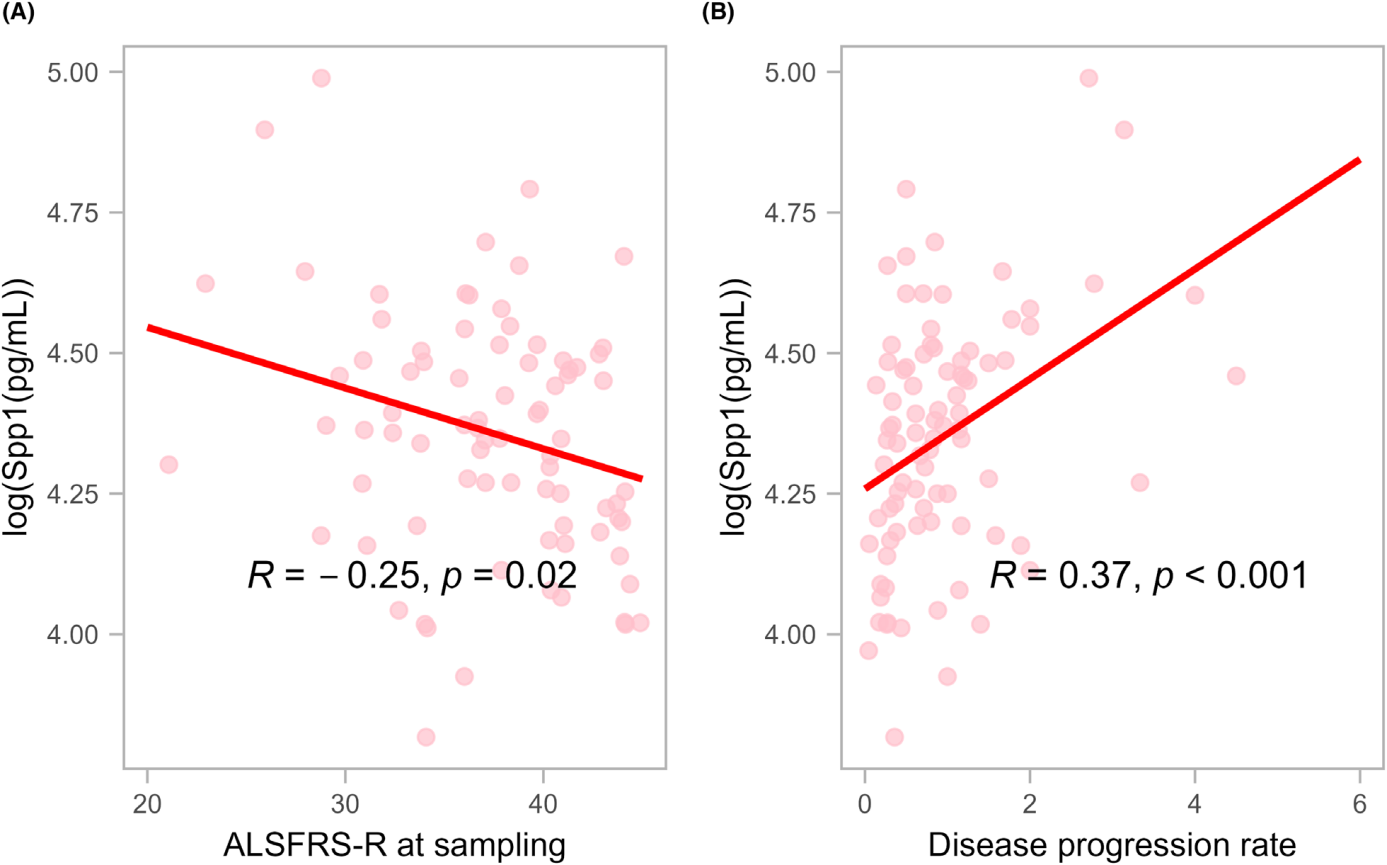
Correlations between serum Spp1 concentrations and clinical parameters of ALS. (A) Correlation between serum Spp1 and ALSFRS‐R score at sampling (*r* = −0.25, *p* = 0.02). (B) Between serum Spp1 and disease progression rate (*r* = 0.37, *p* < 0.001). ALS, amyotrophic lateral sclerosis; ALSFRS‐R, ALS Function Rating Scale‐Revised; Spp1, secreted phosphoprotein 1.

In patients with ALS, an inverse correlation was found between Spp1 concentrations and ALSFRS‐R scores (*r* = −0.25, *p* = 0.02; Fig. 1A); however, after adjusting for age at sampling and sex as covariates, the significance disappeared (*r* = −0.14, *p* = 0.21; Table S5). In patients with SBMA, no significant correlation was observed between Spp1 concentrations and ALSFRS‐R scores (*r* = 0.15, *p* = 0.51).

#### Disease Progression Rate

In patients with ALS, a positive correlation was found between Spp1 concentrations and the DPR (*r* = 0.37, *p* < 0.001; Fig. 1B). After adjusting for age at sampling and sex as a covariate, the partial correlation coefficient remained significant (*r* = 0.31, *p* < 0.01; Table S5). A weak negative correlation was observed between Spp1 concentrations and disease duration (*r* = −0.27, *p* < 0.05). In contrast, no significant correlations were found between Spp1 concentrations and either DPR (*r* = 0.11, *p* = 0.61) or disease duration (*r* = −0.03, *p* = 0.89) in patients with SBMA.

### Longitudinal changes in serum Spp1 levels

The concentrations of serum Spp1 were measured in 23 longitudinal samples from 11 patients with ALS (Table S6 and Fig. S2). The sampling times included the time of diagnosis (baseline, *n* = 11), ≥6 and <12 months after diagnosis (*n* = 6), and ≥12 months after diagnosis (*n* = 6). We tracked the Spp1 concentrations alongside disease progression, as indicated by the declines in ALSFRS‐R scores. Eight patients showed an increasing trend over time, particularly at 12 months or more following diagnosis, while three other patients exhibited a slight decrease or stable course of Spp1 levels.

### Serum Spp1 as a prognostic biomarker

**Figure 2:**
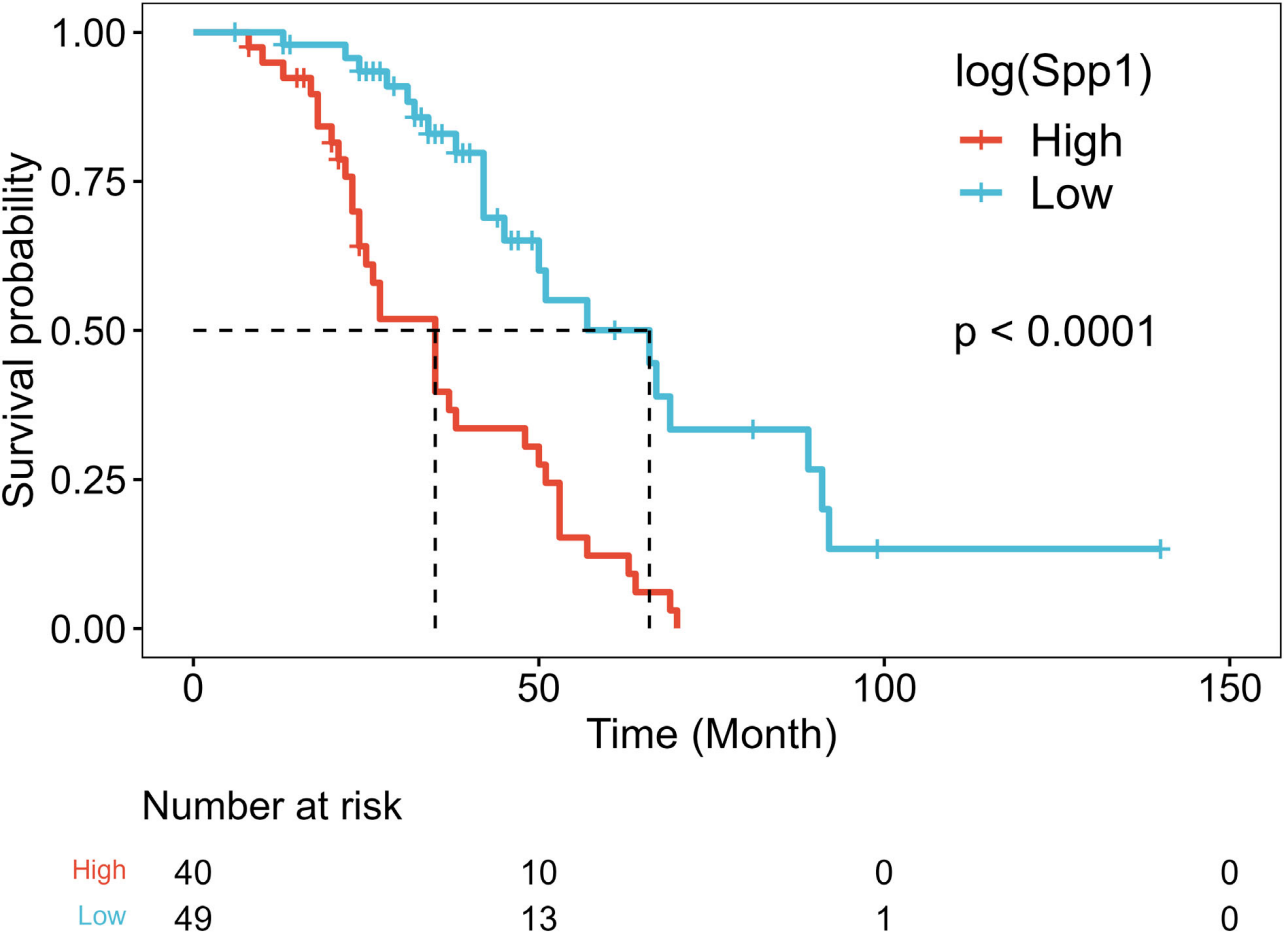
Kaplan–Meier curves for survival in ALS patients. The survival rate was significantly lower in patients with high Spp1 concentrations than in those with low Spp1 concentrations (*p* < 0.001), with a cutoff value for Spp1 set at 24.03 ng/mL. The functional endpoint was defined as death or tracheostomy with invasive ventilation. ALS, amyotrophic lateral sclerosis; Spp1, secreted phosphoprotein 1.

Kaplan–Meier curves showed a significant difference in overall survival rates according to the Spp1 level (*p* < 0.001) (Fig. 2). In univariable Cox regression analysis, the hazard ratio (HR) of log(Spp1) was 27.2 (95% confidence interval [CI], 6.74–109.8, *p* < 0.001) (Table 3). After adjusting for age at onset, sex, BMI, diagnostic delay, ALSFRS‐R, DPR, and onset region, log(Spp1) was found to be an independent prognostic factor for survival (HR, 13.65; 95% CI, 2.57–72.53; *p* < 0.01) (multivariable model 1 in Table 3). Considering FVC as an additional covariate in multivariable model 2, log(Spp1) remained an independent prognostic factor (HR, 16.33; 95% CI, 2.89–92.53; *p* < 0.01).

**Table 3:** Prognostic factors for survival in ALS patients.

	Univariable		Multivariable model 1		Multivariable model 2	
	HR (95% CI)	*p*‐Value	HR (95% CI)	*p*‐Value	HR (95% CI)	*p*‐Value
Sex (female)	0.56 (0.33–0.96)	*	0.62 (0.33–1.18)	0.14	1.65 (0.33–1.29)	0.21
Age at onset	1.04 (0.95–1.01)	**	1.00 (0.96–1.04)	0.92	1.01 (0.97–1.06)	0.52
Age at diagnosis	1.03 (0.97–1.06)	0.67				
BMI (kg/m^2^)	0.92 (0.84–1.01)	0.10	1.08 (0.81–1.03)	0.15	0.93 (0.82–1.04)	0.20
Diagnostic delay	0.94 (0.91–0.97)	***	0.97 (0.94–1.01)	0.12	0.98 (0.94–1.01)	0.16
ALSFRS at diagnosis	0.97 (0.92–1.02)	0.27	1.05 (0.97–1.15)	0.20	1.08 (0.99–1.18)	0.10
DPR	1.80 (1.45–2.34)	***	1.38 (0.84–2.25)	0.20	1.34 (0.81–2.20)	0.24
Onset region (bulbar)	1.45 (0.80–2.61)	0.22	1.46 (0.65–3.28)	0.36	1.45 (0.59–3.55)	0.41
log(Spp1)	27.2 (6.74–109.8)	***	13.65 (2.57–72.53)	**	16.33 (2.89–92.28)	**
FVC (%)					0.99 (0.98–1.01)	0.25

The functional endpoint was defined as death or tracheostomy with invasive ventilation. Statistical analysis methods: Cox proportional–hazards regression.

ALS, amyotrophic lateral sclerosis; ALSFRS‐R, ALS Functional Rating Scale‐Revised; BMI, body mass index; CI, confidence interval; DPR, disease progression rate; FVC, forced vital capacity; HR, hazard ratio; Spp1, secreted phosphoprotein 1.

* *p* < 0.05;

** *p* < 0.01;

*** *p* < 0.001.

## Discussion

In this study, we investigated systemic inflammatory profiles, including serum Spp1, in patients with two distinct motor neuron diseases, ALS and SBMA. Patients with ALS exhibited low‐grade systemic inflammation, whereas those with SBMA showed no clear signs of inflammation. Additionally, we observed that elevated Spp1 levels were significantly correlated with a rapid progression of ALS; however, this trend was not observed in patients with SBMA. Lastly, high Spp1 levels were identified as an independent prognostic factor associated with reduced survival. These findings imply that serum Spp1 could serve as a reliable prognostic biomarker reflecting the extent of neuroinflammation in ALS patients.

Spp1 is a matrix phosphoprotein expressed in various human tissues and cells, including the nervous and immune systems.^18^ It is known to play crucial roles in cell‐mediated immune responses, such as T‐cell differentiation toward T helper 1 (Th1) and Th17 cells, while suppressing Th2 cell development.^19^, ^20^ In addition to its cytokine function,^21^ Spp1 is also involved in inflammatory cell migration and adhesion by interacting with integrin αvβ2 and/or CD44 in most astrocytes and some microglia.^22^, ^23^ In the context of ALS, Spp1 expression has been found to be significantly elevated in microglia in the spinal cord. Interestingly, this upregulation has been observed not only in early‐to‐late stages of ALS rat^24^ and mice^25^, ^26^ models but also during the presymptomatic stage of ALS mice.^14^ A recent study suggested that Spp1‐associated vascular remodeling by perivascular fibroblasts may precede microglial responses and predict the survival of ALS patients.^14^ These findings suggest that Spp1 may contribute to the acceleration of neuronal death through T‐cell‐ and glial cell‐mediated neuroinflammation from the early stages of ALS.

Previous studies have consistently reported elevated Spp1 levels in patients with ALS, compared to healthy controls, both in blood^14^, ^15^ and CSF.^27^, ^28^ In our study, we observed higher levels of serum Spp1 in ALS patients compared to those in the SBMA or the control group, and this difference was only significant in male patients. It is noteworthy that serum and CSF Spp1 levels were shown to be elevated in other neurodegenerative disorders, such as Alzheimer disease^13^, ^15^ and Parkinson disease,^29^ suggesting that Spp1 may not function as a disease‐specific diagnostic biomarker for ALS. Nonetheless, within our ALS cohort, serum Spp1 levels were significantly associated with rapid progression of ALS. Furthermore, Spp1 levels tended to increase as the disease progressed in a subset of patients. This finding may reflect the disease burden of ALS, although the results are limited by the small sample size and thus should thus be interpreted with caution. Finally, elevated Spp1 levels were identified as an independent poor prognostic biomarker for survival. Therefore, Spp1 may serve as a reliable serum prognostic biomarker for disease progression and survival, although its utility as a diagnostic biomarker is limited.

In line with a previous study,^30^ patients with ALS exhibited higher ESR, NLR, and CRP levels than those observed in healthy controls. However, contrary to our expectations, we did not observe a significant correlation between serum Spp1 and these systemic inflammatory markers. This lack of relationship may possibly be attributed to the multifunctional biological nature of Spp1. Typically, blood Spp1 expression is upregulated in relatively common chronic inflammatory conditions, including cardiovascular diseases such as stroke^31^ and peripheral artery disease,^32^ diabetes mellitus,^33^ and hypertension.^34^ While we excluded patients under conditions that could influence Spp1 levels, such as those undergoing immunotherapy or experiencing infection, the complex comorbidities of our patients might have introduced confounding variables into the results. Additionally, there were no significant associations between serum Spp1 and pro‐ or anti‐inflammatory cytokine levels. This preliminary finding may be attributed to the small sample size, underscoring the need for further investigation.

Unlike ALS, no significant changes in systemic inflammatory markers, including serum Spp1, were observed in patients with SBMA, except for the WBC count. The WBC count, NLR, ESR, and CRP level are nonspecific biomarkers of systemic inflammation that can be elevated in infectious, autoimmune, or neurodegenerative diseases where the innate or adaptive immune systems are dysregulated. The modest alteration of systemic inflammatory markers in SBMA compared to ALS suggests a lesser role for inflammation in disease progression than in ALS. Moreover, interestingly, recent studies have shown that axonal damage markers such as neurofilament light^35^ and heavy chains^36^ in the CSF were not elevated in patients with SBMA. These findings may be attributed to the slow progression of motor neuronal death or a lesser contribution of neuroinflammatory response in disease pathogenesis than in typical ALS. Given our results and the consistent finding of prominent CK elevation and reduced creatinine levels in patients with SBMA, primary degenerative changes in skeletal muscles may play a crucial role in the pathogenesis of SBMA, but it seems that released myokines do not have a significant impact on peripheral neuroinflammation.

This study has several limitations. First, the age at blood sampling differed significantly between the SBMA and ALS groups. Based on a nationwide cohort study, the mean age at diagnosis of SBMA was approximately 10 years younger^37^ than that of typical ALS patients^38^; thus, the age difference between the groups was unavoidable. Furthermore, serum Spp1 levels tend to increase with age.^39^ To address this issue, we applied age adjustment in all statistical analyses, including ANCOVA and multivariable linear regression. Second, female controls were not included in the study participants. To ensure reliability, we conducted a sensitivity analysis for male subjects only, resulting in consistent data (see Tables S2 and S3). Third, to assess the clinical severity of SBMA, we utilized the ALSFRS‐R instead of the SBMA Functional Rating Scale because our center has just started using this scale and could not collect retrospective data. Fourth, we did not measure Spp1 in the CSF, which may better reflect intrathecal neuroinflammatory responses.

In conclusion, there is no clear evidence of systemic inflammation in patients with SBMA, suggesting a lesser contribution of neuroinflammation to the pathogenesis of SBMA compared to ALS. Serum Spp1 levels are positively associated with disease progression rate and could be a reliable prognostic biomarker for ALS survival. These findings expand our understanding of these two distinct motor neuron disorders and offer a useful biomarker of neuroinflammation, warranting further investigation.

## Funding Information

This work was supported by the National Research Foundation of Korea (2018R1A5A2025964, 2020R1C1C1005122).

## Conflict of Interest

The authors report no disclosures or conflicts of interest concerning the research related to this manuscript.

## Author Contributions

Concept and design: WHJ, S‐JC, J‐JS. Experiment: J‐JB, JWS, H‐RI, SHK. Acquisition, analysis or interpretation of data: WHJ, S‐JC. Drafting of the manuscript: WHJ, S‐JC. Supervision: Y‐HH, J‐JS, S‐JC. Critical revision of the manuscript for important intellectual content: WHJ, S‐JC, YGM.

## Supporting information


Appendix S1.

## Data Availability

Anonymized data can be obtained upon reasonable request from any qualified investigator for the purpose of replicating procedures and results.
