# Supplementary material for: Association of serum Spp1 levels with disease progression in ALS and SBMA
Source: Ann Clin Transl Neurol. 2024 May 22;11(7):1809–18. doi: 10.1002/acn3.52087 (PMC11251464; doi:10.1002/acn3.52087)
Supplement: Supplementary file 1 — Appendix S1. [file ACN3-11-1809-s001.docx]

Supplemental Material

**Association of serum Spp1 levels with disease progression in ALS and SBMA**

**6 Supplementary Tables**

**Supplementary Table 1.** Comparisons of Laboratory Biomarkers Among Study Groups (Multivariable linear regression)

**Supplementary Table 2.** Comparisons of Laboratory Biomarkers in Male Subjects (ANCOVA)

**Supplementary Table 3.** Comparisons of Laboratory Biomarkers in Male Subjects (Multivariable linear regression)

**Supplementary Table 4.** Comparisons of Serum Cytokine Levels Among Study Groups

**Supplementary Table 5.** Correlations Between Serum Spp1 Levels and Clinical Variables in ALS Patients

**Supplementary Table 6.** Longitudinal Changes of ALSFRS-R Scores and Serum Spp1 Levels in ALS Patients

**2 Supplementary Figures**

**Supplementary Figure 1.** Flow Diagram for Patient Selection

**Supplementary Figure 2.** Longitudinal Changes in Serum Spp1 Concentrations in ALS Patients

**Supplementary Table 1.** Comparisons of Laboratory Biomarkers Among Study Groups (Multivariable linear regression)

|  | **ALS** | **SBMA** | **Control** | *β* ***(P*-value) for Regression Analysis** | |
| --- | --- | --- | --- | --- | --- |
|  |  |  |  | **ALS vs. Control** | **SBMA vs. Control** |
| Age at sampling (years)*^a^* | 58.6±10.8 (n=105) | 54.1±9.9 (n=77) | 57.9±10.6 (n=34) |  |  |
| WBC (10³/μL)*^b^* | 6.86 (5.80-8.49) | 6.29 (5.52-8.00) | 5.34 (4.87-6.37) | 0.18 (0.06) | 0.11 (0.29) |
| NLR*^b^* | 2.13 (1.61-2.90) | 1.46 (1.18-1.98) | 1.69 (1.28-2.23) | 0.23 (0.35) | -0.24 (0.38) |
| CRP (mg/dL)*^b^* | 0.09 (0.04-0.20) | 0.03 (0.01-0.11) | 0.06 (0.02-0.12) | 1.02 (*) | 0.14 (0.76) |
| ESR (mm/h)*^b^* | 16 (8-24) | 9 (4-14) | 8 (5-16) | 1.13 (***) | 0.66 (0.06) |
| CK (U/L)*^b^* | 200 (123-344) | 816 (488-1497) | 183 (104-207) | 0.14 (0.64) | 1.17 (***) |
| Cr (mg/dL)*^b^* | 0.71 (0.60-0.80) | 0.60 (0.55-0.77) | 0.90 (1.81-1.00) | -0.29 (***) | -0.37 (***) |
| Age at sampling (years)*^a^* | 58.9±10.3 (n=88) | 54.9±10.8 (n=28) | 58.3±12.3 (n=31) |  |  |
| Spp1 (ng/mL)*^b^* | 23.08 (15.85-30.65) | 20.90 (16.18-29.53) | 20.46 (13.80-25.90) | 0.15 (0.11) | 0.18 (0.11) |

^a^Data are expressed as mean ± SD. *^b^*Data are expressed as median (interquartile range).

Statistical analysis methods: Multivariable linear regression (covariate: age at sampling). All biomarkers have been transformed using the logarithmic method for analysis. p<0.05* p<0.01** p<0.001***

*Abbreviations:* ALS, amyotrophic lateral sclerosis; CK, creatine kinase; Cr, creatinine; CRP, C-reactive protein; ESR, erythrocyte sedimentation rate; NLR, neutrophil-to-lymphocyte ratio; SBMA, spinobulbar muscular atrophy; Spp1, secreted phosphoprotein 1; WBC, white blood cell

**Supplementary Table 2**. Comparisons of Laboratory Biomarkers in Male Subjects (ANCOVA)

|  | **ALS-Male** | **SBMA** | **Control** | ***P*-value for ANCOVA** | | | |
| --- | --- | --- | --- | --- | --- | --- | --- |
|  |  |  |  | **Overall** | ***Post-hoc* analysis** | | |
|  |  |  |  |  | **ALS vs. Control** | **SBMA vs. Control** | **ALS vs.**  **SBMA** |
| Age at sampling (years)*^a^* | 58.6±10.8 (n=70) | 54.1±9.9 (n=77) | 57.9±10.6 (n=34) | * | 0.92 | * | 0.09 |
| WBC (10³/μL)*^b^* | 6.99 (5.81-8.65) | 6.29 (5.52-8.00) | 5.34 (4.87-6.37) | *** | *** | * | 0.14 |
| NLR*^b^* | 2.36 (1.82-3.33) | 1.46 (1.18-1.98) | 1.69 (1.28-2.23) | *** | ** | 0.56 | *** |
| CRP (mg/dL)*^b^* | 0.08 (0.04-0.19) | 0.03 (0.01-0.11) | 0.06 (0.02-0.12) | *** | 0.17 | 0.66 | ** |
| ESR (mm/h)*^b^* | 14 (8-21) | 9 (4-14) | 8 (5-16) | * | 0.14 | 0.99 | 0.09 |
| CK (U/L)*^b^* | 248 (171-467) | 816 (488-1497) | 183 (104-207) | *** | * | *** | *** |
| Cr (mg/dL)*^b^* | 0.74 (0.64-0.82) | 0.60 (0.55-0.77) | 0.90 (1.81-1.00) | *** | *** | *** | *** |
| Age at sampling (years)*^a^* | 58.5±10.2 (n=56) | 54.9±10.8 (n=28) | 58.3±12.3 (n=31) | 0.33 |  |  |  |
| Spp1 (ng/mL)*^b^* | 24.02 (17.79-31.49) | 20.90 (16.18-29.53) | 20.46 (13.80-25.90) | * | * | 0.21 | 0.88 |

^a^Data are expressed as mean ± SD. *^b^*Data are expressed as median (interquartile range).

Statistical analysis methods: ANOVA test for age at sampling and ANCOVA test for biomarkers (covariate: age at sampling) were used. Tukey method was used for comparing a family of three estimates. All biomarkers have been transformed using the logarithmic method for analysis. p<0.05* p<0.01** p<0.001***

*Abbreviations:* ALS, amyotrophic lateral sclerosis; CK, creatine kinase; Cr, creatinine; CRP, C-reactive protein; ESR, erythrocyte sedimentation rate; F, female; M, male; NLR, neutrophil-to-lymphocyte ratio; SBMA, spinobulbar muscular atrophy; Spp1, secreted phosphoprotein 1; WBC, white blood cell

**Supplementary Table 3.** Comparisons of Laboratory Biomarkers in Male Subjects (Multivariable linear regression)

|  | **ALS-Male** | **SBMA** | **Control** | *β* ***(P*-value) for Regression Analysis** | |
| --- | --- | --- | --- | --- | --- |
|  |  |  |  | **ALS vs. Control** | **SBMA vs. Control** |
| Age at sampling (years)*^a^* | 58.6±10.8 (n=70) | 54.1±9.9 (n=77) | 57.9±10.6 (n=34) |  |  |
| WBC (10³/μL)*^b^* | 6.99 (5.73-8.59) | 6.29 (5.52-8.00) | 5.34 (4.87-6.37) | 0.20 (*) | 0.11 (0.29) |
| NLR*^b^* | 2.36 (1.81-3.35) | 1.46 (1.18-1.98) | 1.69 (1.28-2.23) | 0.44 (0.06) | -0.22 (0.40) |
| CRP (mg/dL)*^b^* | 0.07 (0.04-0.18) | 0.03 (0.01-0.11) | 0.06 (0.02-0.12) | 0.88 (*) | 0.14 (0.74) |
| ESR (mm/h) | 164 (8-21) | 9 (4-14) | 8 (4.5-15.5) | 1.01 (**) | 0.66 (0.08) |
| CK (U/L)*^b^* | 247.5 (170.75-467) | 815.5 (487.5-1497) | 183 (104-207) | 0.41 (0.19) | 1.118 (***) |
| Cr (mg/dL)*^b^* | 0.74 (0.64-0.82) | 0.6 (0.55-0.77) | 0.9 (1.81-1.00) | -0.21 (**) | -0.37 (***) |
| Age at sampling (years)*^a^* | 58.1±9.9 (n=56) | 54.9±10.8 (n=28) | 58.3±12.3 (n=31) |  |  |
| Spp1 (ng/mL)*^b^* | 24.02 (17.79-31.49) | 21.47 (17.22-29.53) | 20.46 (15.16-26.15) | 0.10 (*) | 0.08 (0.09) |

^a^Data are expressed as mean ± SD. *^b^*Data are expressed as median (interquartile range).

Statistical analysis methods: Multivariable linear regression (covariate: age at sampling). All biomarkers have been transformed using the logarithmic method for analysis. p<0.05* p<0.01** p<0.001***

*Abbreviations:* ALS, amyotrophic lateral sclerosis; CK, creatine kinase; Cr, creatinine; CRP, C-reactive protein; ESR, erythrocyte sedimentation rate; F, female; M, male; NLR, neutrophil-to-lymphocyte ratio; SBMA, spinobulbar muscular atrophy; Spp1, secreted phosphoprotein 1; WBC, white blood cell

**Supplementary Table 4.** Comparisons of Serum Cytokine Levels Among Study Groups

|  | **ALS (n=14)** | **SBMA (n=15)** | **Control (n=10)** | ***P*-value** |
| --- | --- | --- | --- | --- |
| IFN-$\gamma$ (ng/mL) | 6.31 (4.98-8.26) | 5.26 (4.51-8.78) | 6.12 (4.72-15.8) | 0.68 |
| IL-10 (ng/mL) | 0.23 (0.17-0.47) | 0.26 (0.21-0.38) | 0.24 (0.21-0.39) | 0.89 |
| IL-1$\beta$ (ng/mL) | 0.09 (0.04-0.13) | 0.06 (0.04-0.12) | 0.10 (0.08-0.13) | 0.54 |
| IL-6 (ng/mL) | 0.79 (0.56-8.41) | 1.31 (0.78-1.47) | 1.00 (0.73-1.14) | 0.58 |
| IL-8 (ng/mL) | 9.77 (7.89-14.7) | 10.3 (9.16-17.0) | 28.0 (23.0-39.0) | 0.03 |
| TNF-$\alpha$ (ng/mL) | 1.41 (1.09-1.81) | 1.39 (1.07-1.60) | 1.17 (1.08-1.50) | 0.55 |
| IL-17$\alpha$ (ng/mL) | 1.38 (0.78-1.99) | 1.13 (0.78-1.56) | 0.70 (0.54-1.58) | 0.66 |

Data are expressed as median (interquartile range). Statistical analysis methods: Kruskal-Wallis test.

*Abbreviations:* ALS, amyotrophic lateral sclerosis; IFN-$\gamma$, interferon gamma; IL, interleukin; SBMA, spinobulbar muscular atrophy; TNF-$\alpha$, tumor necrosis factor alpha

**Supplementary Table 5.** Correlations Between Serum Spp1 Levels and Clinical Variables in ALS Patients

|  | **Serum Spp1** | | | |
| --- | --- | --- | --- | --- |
|  | Correlation coefficient | | Partial correlation coefficient  (Controlling the effects of Age and Sex) | |
|  | r | *P*-value | r | *P*-value |
| ALSFRS-R at sampling | -0.25 | * | -0.14 | 0.21 |
| DPR | 0.37 | *** | 0.31 | ** |

Statistical analysis methods: Spearman correlation test. p<0.05* p<0.01** p<0.001***

*Abbreviations:* ALS, amyotrophic lateral sclerosis; ALSFRS-R, ALS functional rating scale-revised; DPR, disease progression rate; Spp1, secreted phosphoprotein 1

**Supplementary Table 6.** Longitudinal Changes of ALSFRS-R Scores and Serum Spp1 Levels in ALS Patients

|  | | **Baseline** | **6m** $\boldsymbol{\leq}$ **F/U < 12m** | **F/U** $\boldsymbol{\geq}$ **12m** |
| --- | --- | --- | --- | --- |
| **Patient 1** | ALSFRS-R | 32 |  | 25 |
|  | Spp1 (ng/mL) | 24.75 |  | 43.46 |
| **Patient 2** | ALSFRS-R | 45 |  | 32 |
|  | Spp1 (ng/mL) | 11.08 |  | 16.31 |
| **Patient 3** | ALSFRS-R | 40 |  | 36 |
|  | Spp1 (ng/mL) | 18.12 |  | 25.44 |
| **Patient 4** | ALSFRS-R | 45 | 39 |  |
|  | Spp1 (ng/mL) | 40.35 | 31.50 |  |
| **Patient 5** | ALSFRS-R | 21 | 14 |  |
|  | Spp1 (ng/mL) | 20.03 | 18.91 |  |
| **Patient 6** | ALSFRS-R | 45 | 36 | 26 |
|  | Spp1 (ng/mL) | 27.74 | 28.15 | 71.00 |
| **Patient 7** | ALSFRS-R | 34 | 29 |  |
|  | Spp1 (ng/mL) | 30.50 | 32.70 |  |
| **Patient 8** | ALSFRS-R | 33 | 29 |  |
|  | Spp1 (ng/mL) | 29.32 | 35.63 |  |
| **Patient 9** | ALSFRS-R | 44 |  | 35 |
|  | Spp1 (ng/mL) | 17.07 |  | 32.10 |
| **Patient 10** | ALSFRS-R | 40 |  | 27 |
|  | Spp1 (ng/mL) | 19.82 |  | 33.54 |
| **Patient 11** | ALSFRS-R | 44 | 38 |  |
|  | Spp1 (ng/mL) | 17.93 | 17.12 |  |

*Abbreviations:* ALS, amyotrophic lateral sclerosis; ALSFRS-R, ALS functional rating scale-revised; F/U, follow-up; Spp1, secreted phosphoprotein 1

**Supplementary Figure 1.** Flow Diagram for Patient Selection (upper panel: ALS, lower panel: SBMA)

**SBMA patients**

in SNUH MND registry

(Between Jan 2002 and Dec 2023, n=177)

Immunotherapy (n=13)

(Steroid = 10; IVIg = 3)

**Study inclusion** (n=77 M/F=77/0)

History of

Infection (n=5), Cancer (n=6),

Rheumatoid/Autoimmune disease (n=1)

(n=1)

Insufficient data (n=75)

**ALS patients**

in SNUH MND registry

(Between Feb 2017 and Aug 2023, n=596)

Immunotherapy (n=153)

(Steroid = 84; IVIg = 103; Steroid & IVIg = 43; RTX = 9)

Insufficient data (n=279)

History of

Infection (n=35), Cancer (n=16),

Rheumatoid/Autoimmune disease (n=8)

**Study inclusion (**n=105, M/F=70/35)

*Abbreviations:* ALS, amyotrophic lateral sclerosis; IVIg, intravenous immunoglobulin; MND, motor neuron disease; RTX, rituximab; SBMA, spinobulbar muscular atrophy; SNUH, Seoul National University Hospital

**Supplementary Figure 2.** Longitudinal Changes in Serum Spp1 Concentrations in ALS Patients


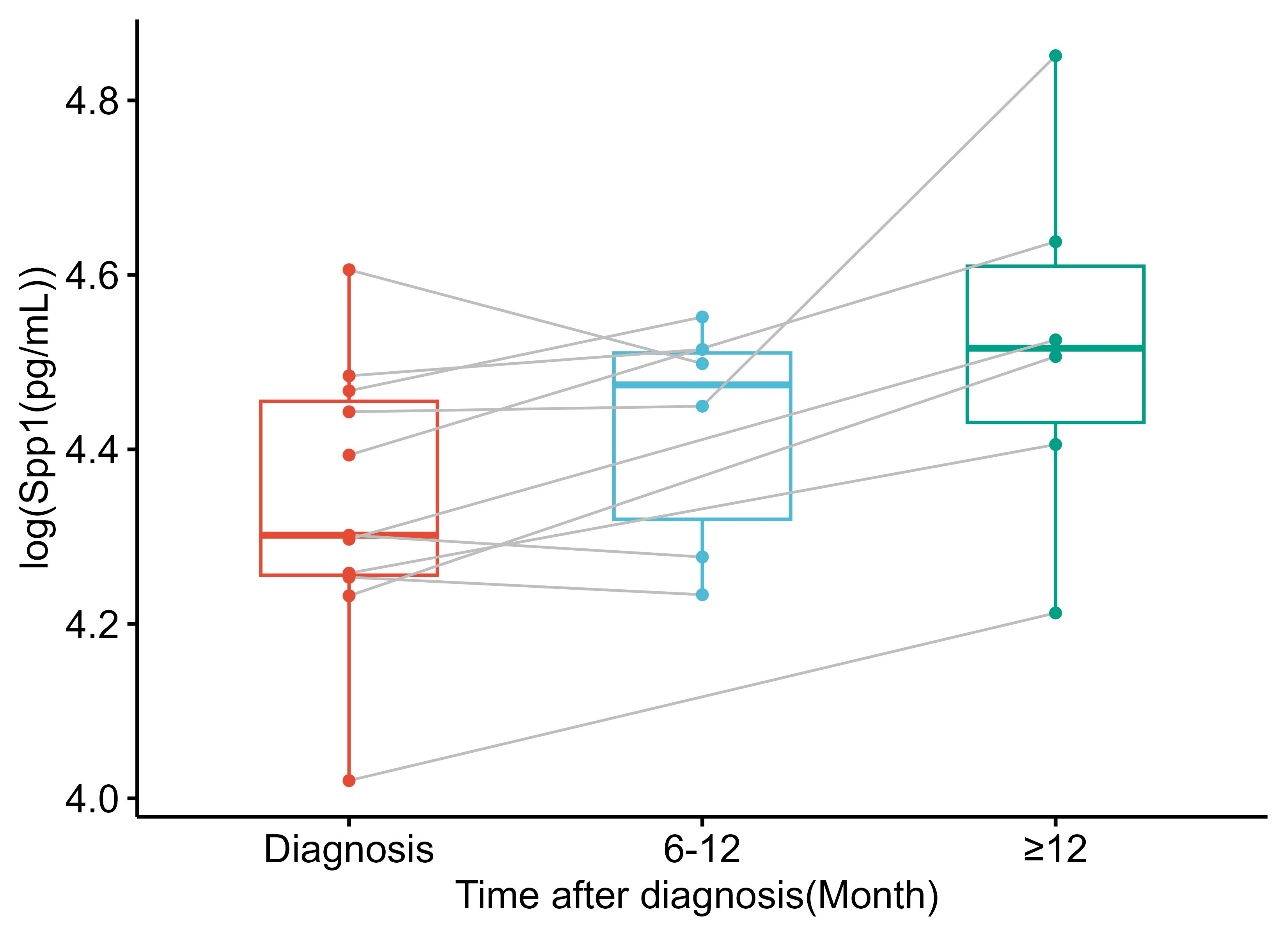


Serum Spp1 levels measured at the time of diagnosis (n = 11), from 6 to 12 months after diagnosis (n = 6), and those after diagnosis ≥ 12 months (n = 6) are depicted.

*Abbreviations:* ALS, amyotrophic lateral sclerosis; Spp1, secreted phosphoprotein 1
